# Bacterial Fluorinase FIA1 and Evolutionarily Related, Lysine-free StDUF62 Show Distinct Diastereoselectivity and Salt Sensitivity

**DOI:** 10.1021/acsomega.5c00855

**Published:** 2025-05-15

**Authors:** Andrej Tekel, Martin Orságh, Martin Dračínský, Tomáš Pluskal

**Affiliations:** † Institute of Organic Chemistry and Biochemistry of the Czech Academy of Sciences, Flemingovo náměstí 542/2, 160 00 Prague 6, Czech Republic; ‡ Department of Physical and Macromolecular Chemistry, Faculty of Science, 112302 Charles University, Albertov 6, 120 00 Prague 2, Czech Republic

## Abstract

*S*-adenosyl-l-methionine (SAM) is a crucial enzymatic cofactor that is conserved across all domains of life. Despite the pivotal role of this cofactor, its chirality at the sulfonium sulfur and the differing biological activities of its epimers, (*S*,*S*)-SAM and (*R*,*S*)-SAM, are often overlooked. Although enzymes predominantly utilize the (*S*,*S*)-SAM epimer, due to spontaneous epimerization at the sulfonium sulfur of SAM, the (*R*,*S*)-SAM epimer is present in all cells as well as in commercial SAM-containing products. Recently, an enzyme containing the DUF62 domain, identified as Salinispora tropica (StDUF62), has been shown to selectively hydrolyze (*R*,*S*)-SAM. It has been hypothesized that this function prevents the problematic accumulation of this epimer. Fluorinases, the only enzymes known to naturally incorporate fluorine into organic compounds, are homologous to enzymes of the DUF62 family. The discovery of unexpected diastereoselectivity of StDUF62 however raised an important question regarding the diastereoselectivity of the evolutionarily related bacterial fluorinase FlA1, an enzyme of significant importance. Given the relationship between these enzymes and their similar catalytic functions, it would be reasonable to hypothesize that FlA1 might also demonstrate activity toward the (*R*,*S*)-SAM diastereomer. Despite this homology, we report here the opposite diastereoselectivity of StDUF62 and Streptomyces sp. MA37 fluorinase (FlA1). The unusual lysine-free amino acid composition of StDUF62 suggests an evolutionary origin in haloadaptation; however, its SAM-hydrolyzing activity is greatly diminished at physiological concentrations of KCl or NaCl. We show that this inhibition is not caused solely by the competition with the chloride anion, as Na_2_SO_4_ at equivalent ionic strength is also greatly diminishing StDUF62 activity, contrary to the fluorinating activity of FlA1. Both adenosine and increased ionic strength promoted StDUF62 trimer formation, whereas increased ionic strength alone led to inhibition. Considering the contrast between the wasteful hydrolysis of (*R*,*S*)-SAM and the energetically efficient mechanisms of eukaryotic (*R*,*S*)-SAM recycling, we suggest that (*R*,*S*)-SAM hydrolysis might not be the physiological function of StDUF62.

## Graphical abstract

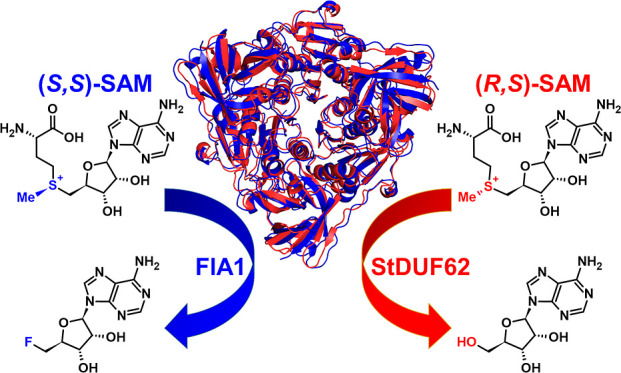

## Introduction

*S*-Adenosyl-l-methionine (SAM) is an omnipresent enzymatic cofactor. Due to its irreplaceable role in cellular metabolism, it is conserved throughout all domains of life. Being an amino acid–nucleotide conjugate and a Rossmann fold-binding cosubstrate/cofactor with unique chemical capabilities, SAM is considered a possible remnant of ancient versions of life.^1,2^ Although SAM is commonly discussed as a methyl group donor utilized by various methyltransferases, its biochemistry is much more diverse,^3^ for example, radical SAM enzymes employ reductive cleavage of SAM to produce typically the 5′-deoxyadenosyl radical that is involved in a vast range of chemical reactions such as C–H bond activations, epimerization of amino acids in peptides, and many more.^4^

It has been widely overlooked in the literature that SAM possesses a center of chirality on the sulfonium sulfur.^5^ However, it is known that the two sulfonium epimers, referred to as (*S*,*S*)-SAM and (*R*,*S*)-SAM, have different biological activities.^6^ The (*S*,*S*)-SAM epimer is the diastereomer that is biosynthesized and used by almost all enzymes, but some enzymes do not differentiate or even prefer (*R*,*S*)-SAM.^7,8^ Since the sulfonium of the (*S*,*S*)-SAM epimer slowly undergoes reversible uncatalyzed pyramidal inversion to (*R*,*S*)-SAM, commercial and in-house made preparations of SAM can contain up to 50% of (*R*,*S*)-SAM.^9^

In 2002, SAM was described as a substrate in the biosynthesis of 5′-deoxy-5′-fluoroadenosine, catalyzed by fluorinases, which are to this date the only enzyme family known to naturally incorporate fluorine into organic compounds.^10^ DUF62 is also a Rossmann fold-derived family of proteins that uses SAM as a substrate. Fluorinases have been compared with DUF62 proteins due to their sequence homology, and it is hypothesized that they could have evolved from this class of enzymes.^11^ Studies on DUF62 enzymes have shown that they catalyze hydrolysis of SAM to methionine and adenosine.^12^ Although fluorinases are utilized in the biosynthetic pathway of fluoroacetate, it has been unknown why there would be a need for DUF62 catalyzing the hydrolysis of SAM back to its precursors, as SAM biosynthesis is energetically exhausting. Recently, DUF62 from Salinispora tropica (further referred to as StDUF62) has been shown to stereoselectively hydrolyze only (*R*,*S*)-SAM.^13^ The relevance of such an enzyme for cellular metabolism was justified by the conjecture that (*R*,*S*)-SAM build-up can be toxic to the cell, and therefore, hydrolysis of (*R*,*S*)-SAM is desirable.^13,14^ The diastereoselectivity of fluorinase enzymes, to the best of our knowledge, has not yet been addressed experimentally. Due to the recently observed rare diastereoselectivity of StDUF62 toward the (*R*,*S*)-SAM epimer and remarkable similarities between the DUF62 family and fluorinases, we have decided to investigate the stereoselectivity of fluorinase FlA1 from Streptomyces sp. MA37 (further referred to as FlA1) and compare it with that of StDUF62.

## Results

To address the diastereoselectivity of StDUF62 and FlA1, we performed an NMR kinetics study (Figure 1A,B), observing the gradual enzymatic conversion of SAM over time manifested as the decrease in the intensity of the characteristic methyl group attached directly to the sulfonium center (Figure 1C).^17^ Concentration of SAM was chosen to approximate the concentrations known to be physiological.^18^ Remarkably, even though the two proteins share sequence homology (42.3% sequence similarity and 27.7% sequence identity) and the predicted structure of StDUF62 very closely resembles the known structure of FlA1 (Figure 1D), we did not observe these two enzymes to show the same diastereoselectivity. As several structures of StDUF62 homologues are already deposited in the PDB, the AlphaFold structure has high prediction confidence (average pLDDT score 96.5 out of 100). Further comparisons with published StDUF62 homologues can be seen in Supporting Information Figure S2. Our measurements reveal that FlA1 exhibits diastereoselectivity opposite to that of StDUF62, preferring the (*S*,*S*)-isomer, while StDUF62 primarily targets the (*R*,*S*)-isomer (Figure 1A). It is also interesting that in our hands, after complete depletion of the (*R*,*S*)-SAM epimer, StDUF62 showed activity toward the (*S*,*S*)-SAM epimer (Figure 1A), although the hydrolysis of the (*S*,*S*)-isomer did not proceed to full completion. The epimerization by sulfonium pyramidal inversion of (*R*,*S*)-SAM to (*S*,*S*)-SAM or vice versa can be neglected on the time scale of our experiments, as the kinetic constant of this transformation is known and is too slow to account for the observed changes (*k*_*r*_ = 1.8 × 10^–6^ s^–1^).^9^

**Figure 1:**
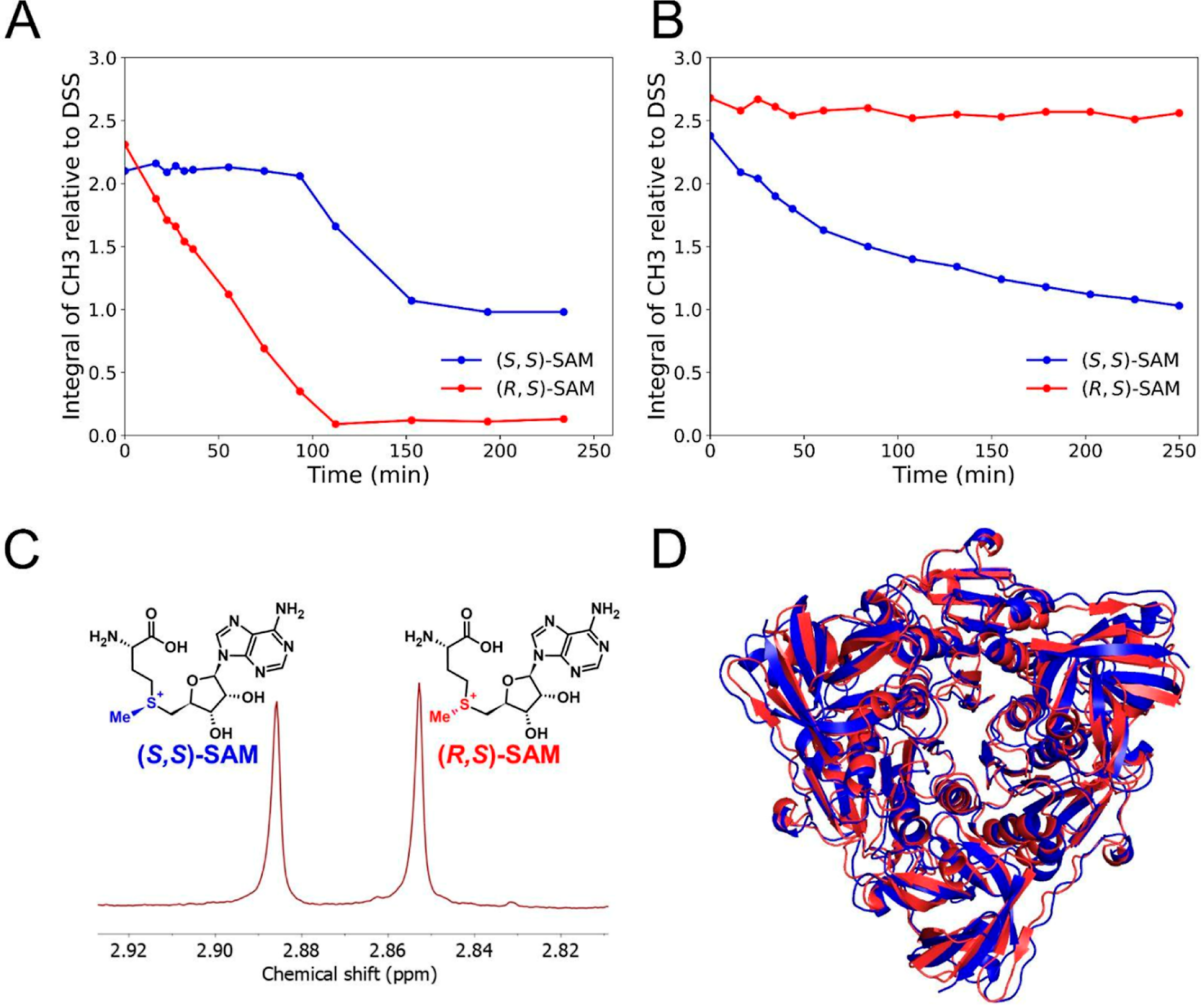
Conversion of a mixture of (*S*,*S*)- and (*R*,*S*)-SAM by (A) StDUF62 and (B) FlA1, as monitored by NMR. Reactions were carried out using 10 μM of FlA1 or StDUF62 with 200 μM racemic SAM in 50 mM phosphate buffer (pH 8), supplemented with 10% D_2_O and 15 μM DSS (sodium trimethylsilylpropanesulfonate) as an internal standard. For FlA1, 20 mM NaF was added. A series of ^1^H NMR spectra was recorded at 303 K. (C) ^1^H NMR-resolved peaks of the sulfonium methyl group of (*S*,*S*)- and (*R*,*S*)-SAM. (D) Experimental trimeric structure of FlA1 (blue; PDB: 5b6i) and superimposed (RMSD 1.7 Å) trimeric AlphaFold structure of StDUF62 (red; AF DB: AF-A4 × 4S2–F1-v4).^15,16^ Molecular graphics were rendered with PyMOL 2.5.8.

While performing bioinformatic analyses, it caught our attention that the StDUF62 enzyme does not contain any lysine residues and is highly enriched in arginine. This enzyme originates from *S. tropica*, a bacterium originally isolated from tropical marine sediments in the Bahamas.^19^ Proteins originating from halophilic and marine organisms are known to be depleted in lysine and enriched in arginine, although this does not apply to all protein families and the extent of this effect is not clear.^20^ First, we investigated whether this amino acid composition is specific to StDUF62 or whether it is a more general property of the DUF62 family.

CD-HIT clustering of Pfam family PF01887, which includes all DUF62 enzymes, revealed several patterns (Figure 2). Clusters 2 and 3 are bacterial clusters, whereas cluster 1 exclusively contains archaeal sequences, thus showing that low-lysine high-arginine sequences are not specific only to bacterial members of this family. While all sequences in the exclusively archaeal cluster 1 belong to halophilic archaea, this is not the case for bacterial clusters 2 and 3. Many DUF62 sequences in these clusters belong to bacteria that are known to live in environments with fairly low salinity, for example, Actinomadura pelletieri, a known human pathogen, orCryptosporangium arvum, a soil bacterium isolated from a vegetable field.^21,22^ Due to the interesting absence of lysine and high arginine content of StDUF62, we decided to examine the effect of KCl and NaCl on this enzyme, as these properties are likely the result of haloadaptation.

**Figure 2:**
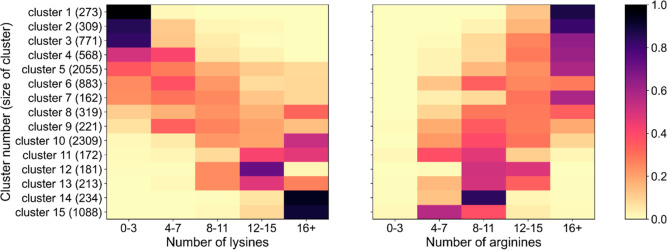
Heatmaps presenting binned counts of lysines and arginines in clusters from CD-HIT analysis of Pfam family PF01887. The family was clustered by CD-HIT to 0.4 sequence similarity. In each cluster, the lysines or arginines in each sequence were counted, and their counts were binned into five bins (0–3, 4–7,8–11,12–15 and 16 or greater). The frequency of each bin in the 15 most populated clusters is presented.

To our surprise, the hydrolytic activity of StDUF62 was largely diminished at only 150 mM NaCl or KCl (Figure 3). The intracellular concentrations of salts in most bacteria have not been studied, but from what is known we understand that potassium is the usual intracellular cation, that chloride is the usual intracellular anion, and that the overall intracellular concentration of salts is expected to be at least in several hundred millimolar range.^23^ Marine bacteria live in an environment with much greater (approximately 600 mM) total salt concentrations than their counterparts living in fresh waters, so they need to compensate for this by higher intracellular salt concentrations or higher concentrations of osmolytes. When the concentration of KCl or NaCl increased to 300 mM, the StDUF62 enzyme lost its hydrolytic activity almost completely, which seems to be an unexpected result for an enzyme found in marine bacteria. Even more surprisingly, in the case of FlA1, which originates from the soil bacterium Streptomyces sp. MA37, the enzyme retains half of its activity under conditions where almost no activity from StDUF62 is observed. Inhibition of FlA1 by NaCl has likely significant competitive contribution, since 300 mM NaF or 100 mM Na_2_SO_4_ does not display inhibition of such magnitude as NaCl (*p* < 0.01, Welch’s *t*-test).

**Figure 3:**
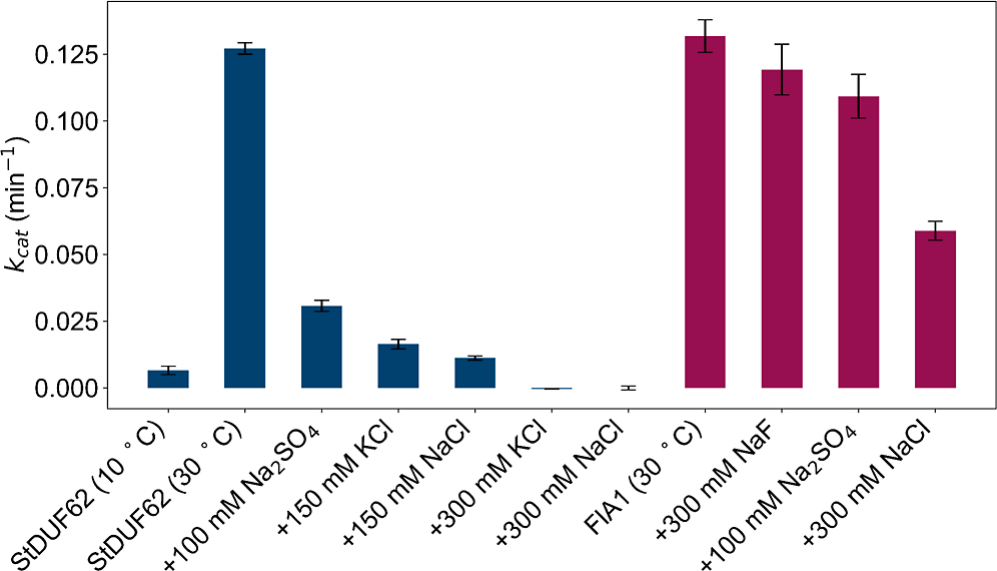
LC–MS kinetics measurements of StDUF62 and FlA1, measured as methionine production (methionine being a common product of FlA1 and StDUF62). 10 μM enzyme (50 μL final volume) with 300 μM of SAM were incubated for 15 min at 30 °C (except for the 10 °C StDUF62 condition) in a 50 mM phosphate buffer at pH 8. For each FlA1 except the 300 mM NaF, 20 mM NaF was added. Measurements for each set of conditions were run in triplicates. The results are presented as means ± SD.

Furthermore, it is interesting to point out that StDUF62 inhibition is not caused simply by chloride competition, as sodium sulfate at equivalent ionic strength (100 mM Na_2_SO_4_ has equal ionic strength as 300 mM NaCl or KCl) is also displaying strong inhibition (Figure 3), albeit smaller than 300 mM KCl or NaCl (*p* < 0.01, Welch’s *t*-test). While we and also authors of previous work were working with StDUF62 at 30 °C,^24^ this is likely not the temperature S. tropica is typically experiencing. It was shown that S. tropica grows even at deep marine sediments at depths of about 1100 m.^24^ Temperature in the oceans drops very rapidly with increasing depth until a body of roughly constant temperature is reached. This body of water has a temperature roughly between 4 to 10 °C.^25^ For this reason, we also measured activity at 10 °C, as we consider it informative for its putative physiological function, but very little activity at this temperature was observed, compared to 30 °C (Figure 3). Since chlorinase is also related to DUF62 enzymes, we checked for the potential formation of 5′-deoxy-5′-chloroadenosine; however, none was being formed (data not shown). The unusual amino acid composition of StDUF62 and its unexpectedly strong inhibition by NaCl and KCl prompted us to examine its solution behavior under assayed conditions.

Although it is known that FlA1 is trimeric in solution and that its trimers partially associate to form hexamers (dimers of trimers),^27^ to our best knowledge, the solution oligomerization properties of StDUF62 or DUF62 have not been studied in general. In the published structures of other DUF62 enzymes and FlA1s, the catalytic site is located at the interface between particular protomers of the trimeric assembly. We therefore hypothesized that the observed inhibitory effect could be explained by the dissolution of the trimer. Analytical ultracentrifugation of StDUF62 (Figure 4A) revealed the value of the sedimentation coefficient normalized to standard conditions, *s*_w(20,w)_, as 3.68 S, which is between the theoretical values for the monomer (2.39 S) and the trimer (4.84 S), calculated using a model from data available in the AlphaFold structural database.^16,28^ This can be interpreted as the formation of dimers or as a fast equilibrium between monomers and trimers. Interestingly, the addition of 300 mM NaCl or 300 μM adenosine pushed the equilibrium toward the trimer (*s*_w(20,w)_ equaling 4.22 and 4.73 S, respectively). The sedimentation profile of FlA1 (Figure 4A) shows distinct peaks for the trimer (*s*_w(20,w)_ = 5.72 S) and hexamer (*s*_w(20,w)_ = 8.96 S), matching the values calculated from the deposited PDB structure (*s*_w(20,w)_ = 5.90 and 9.02 S for the trimer and hexamer, respectively).^15,28^ However, NaCl inhibited the function of StDUF62, whereas adenosine did not markedly alter the enzyme’s activity (Figure 4B) (*p* = 0.103, Welch’s *t*-test). The fact that the presence of both NaCl and adenosine promoted the formation of the trimer, while having the opposing effect on the activity of the enzyme, disproves the hypothesis that the inhibition is caused either by the dissolution or formation of the trimer. We therefore assume that the structural changes causing the inhibitory effect are more subtle.

**Figure 4:**
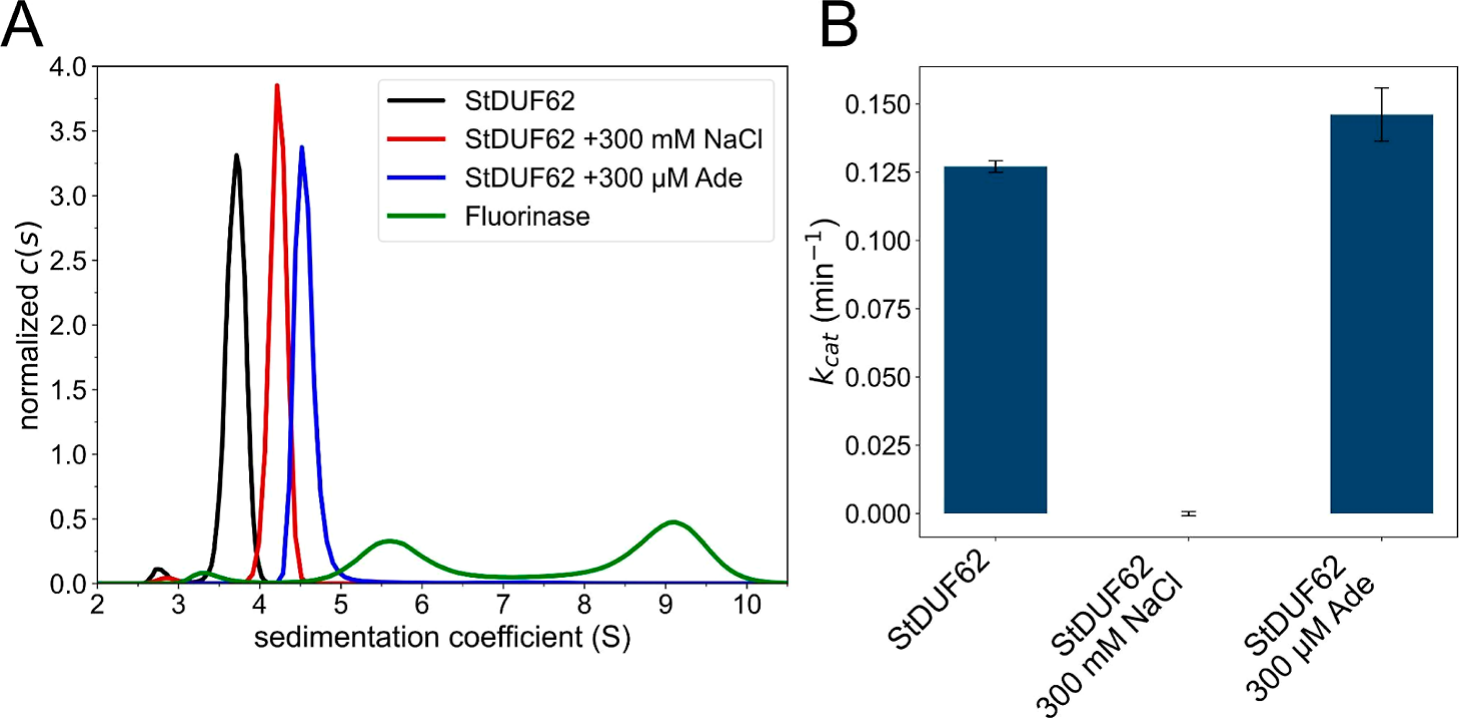
(A) The *c*(*s*) distribution of sedimentation coefficients of StDUF62 and FlA1. The distributions were calculated and transformed to standard conditions using SEDFIT.^26^ (B) LC–MS kinetics measurements of StDUF62, measured as methionine production. 10 μM (50 μL final volume) enzyme with 300 μM of SAM were incubated for 15 min at 30 °C in a 50 mM phosphate buffer at pH 8. Measurements for each set of conditions were run in triplicates. The results are presented as means ± SD.

## Discussion

The different diastereoselectivity of FlA1 and StDUF62 is quite interesting, since the two enzymes belong to the same family of proteins. When we first read the report of Kornfuehrer et al. about the diastereoselectivity of StDUF62 toward the (*R*,*S*)-SAM,^13^ we immediately examined the available structures of fluorinases in the PDB database. While there is no structure of FlA1 with SAM, there were two structures (PDB IDs: 1rqp and 2v7u) of the Streptomyces cattleya fluorinase FlA with the (*S*,*S*)-SAM ligand included in the structure model.^29,30^ However, in both of these structures, 1rqp and 2v7u, the structure model is a poor fit to the electron density map around the sulfonium center of (*S*,*S*)-SAM. While the 2v7u structure comes from a crystal obtained in the presence of SAM, in the case of 1rqp, the ligand was copurified from bacteria. There are many possible causes for the poorly interpretable electron density around the sulfonium center. Some of the typical reasons include inherent flexibility of the ligand, radiation damage, or low diffraction resolution. In the case of 2v7u, we also considered the possibility of an (*S*,*S*)-SAM/(*R*,*S*)-SAM mixture in the crystal (as commercial and in-house preparations of SAM contain both diastereomers). In the case of 1rqp, a possible explanation in accordance with the electron density is that the ligand present in the crystal is actually *S*-adenosyl-l-homocysteine (SAH), as SAH is present in bacterial cells and is known to bind FIA1 with higher affinity than SAM.^30^ The fact that the QM/MM study by Senn et al. also concluded that the (*S*,*S*)-SAM sulfonium methyl conformation in 1rqp is likely artificial^31^ further fueled our hypothesis that fluorinases could be similar in the regard of diastereoselectivity to StDUF62 and prefer the (*R*,*S*)-SAM diastereomer. Nevertheless, this turned out to not be the case, and the diastereoselectivity of FlA1 is opposite to the StDUF62.

While it is true that StDUF62 is able to hydrolyze (*R*,*S*)-SAM, our findings that even low salt concentrations or more typical marine temperatures significantly diminish the activity of StDUF62 cast doubt on whether this hydrolytic activity is indeed a physiological one. Comparing, for example, the known mechanism in eukaryotes that utilizes (*R*,*S*)-SAM-specific methyltransferases to methylate homocysteine,^8,14^ a bacterial/archaeal mechanism that would just hydrolyze (*R*,*S*)-SAM seems like an energetically inefficient pathway. We cannot definitely reject the hypothesis that the physiological role of DUF62 enzymes is to prevent the buildup of (*R*,*S*)-SAM, but our findings suggest that at least some DUF62 enzymes might have another, so far unknown, physiological function.

We can further extend this question to fluorinases. The major fluorometabolites of fluorinase-producing bacteria, fluoroacetate and fluorothreonine, do not possess strong antibiotic activity. Fluoroacetate accumulation is known to occur in some plants, and there it can be easily explained as protection against herbivores, as it exhibits high toxicity toward many mammals.^32^ It is often overlooked that these compounds do not tend to be very toxic toward bacteria or lower eukaryotes. Even though the native environment of soil bacteria (such as Streptomyces sp. MA37) is complex, data on model organisms indicate that up to 6 mM concentrations of fluoroacetate are not lethal for Aspergillus niger^33^ and that Escherichia coli is able to grow in media containing even up to 100 mM of fluoroacetate (“permanent suppression of growth could not be achieved even by raising the concentration of fluoroacetate to 10^–1^ M.”).^34^ These concentrations cannot be reasonably achieved in nature and are outside the range typically observed for antibiotics. This does not refute the possible advantage fluorinase-expressing bacteria might obtain against very specific competitors as the biological environment can be very complex. Nevertheless, it also suggests that we might not have a complete picture about the physiological significance of fluorinases and enzymes of the DUF62 family, from which fluorinases have likely evolved.

## Supplementary Material



## Notes

Materials and methods, DNA and amino acid sequences of FlA1 and StDUF62, SDS-PAGE of StDUF62 and FlA1, and comparison of StDUF62 AlphaFold prediction with known experimental structures from the DUF62 family (PDF)
