# Supplementary material for: Bacterial Fluorinase FIA1 and Evolutionarily Related, Lysine-free StDUF62 Show Distinct Diastereoselectivity and Salt Sensitivity
Source: ACS Omega. 2025 May 15;10(20):20509–14. doi: 10.1021/acsomega.5c00855 (PMC12120602; doi:10.1021/acsomega.5c00855)
Supplement: Supplementary file 1 [file ao5c00855_si_001.pdf]

Supporting Information for

Bacterial fluorinase FIA1 and evolutionarily related, lysine-free StDUF62 show distinct diastereoselectivity and salt sensitivity

Andrej Tekel[1,2], Martin Orságh[1,2], Martin Dračínský[1], Tomáš Pluskal[1*]

[1]Institute of Organic Chemistry and Biochemistry of the Czech Academy of Sciences, Flemingovo náměstí 542/2, 160 00 Praha 6, Czech Republic.

[2]Department of Physical and Macromolecular Chemistry, Faculty of Science, Charles University, Albertov 6, 120 00, Prague 2, Czech Republic.

*Email: tomas.pluskal@uochb.cas.cz

Materials and Methods

Production of fluorinase and StDUF62: Fluorinase from *Streptomyces* sp. MA37 (Uniprot ID: W0W999) in pET-28a(+), with TEV-cleavable N-terminal His-tag was obtained as a gift from Zbynek Prokop (Loschmidt Laboratories, Brno, Czechia). Thrombin site in this plasmid is modified as described in previous work.[1] DUF62 from Salinispora tropica (Uniprot ID: A4X4S2) was synthesized and inserted into pET-28a(+) with TEV-cleavable N-terminal His-tag by Twist Bioscience (TEV site was inserted during gene synthesis). Chemically competent *Escherichia coli* BL21(DE3) were transformed with these plasmids. Purification protocol was the same for both proteins. A single colony was picked and cultured overnight at 37 °C, 200 rpm in 5 mL of Lysogeny broth medium supplemented with 50 µg/ml of kanamycin. The pre-cultured bacteria were then transferred into 0.5 L of Terrific broth medium supplemented with 2 g/L lactose, 2 g/L glycerol and 50 µg/mL kanamycin in an 2 L Erlenmayer flask and grown for 2 hours at 37 °C and 200 rpm. The temperature was subsequently lowered to 20 °C and the culture was left to grow overnight. Cells were then harvested by centrifugation (3000g, 20min) and resuspended in a lysis buffer (1x PBS, 500 mM NaCl, pH 7.5). Lysosyme (100 µg/mL) was added to the suspension, left to incubate for 30 min at 4 °C and subsequently sonicated. Lysate was centrifuged and the supernatant was loaded to Ni-NTA resin. Following washing, protein was eluted with the elution buffer (1x PBS, 500 mM imidazole, 500 mM NaCl, 2 mM BME, pH 8) and dialyzed for two hours against dialysis buffer (1x PBS, 150 mM NaCl, 2 mM BME, pH 7.5). Afterwards, TEV was added and protein was then put into dialysis overnight. After tag cleavage, gel filtration (Superdex 200 10/300) was run into the final buffer (20 mM phosphate buffer, 100 mM NaCl, pH 8). Protein concentration was assessed with a Nanodrop and the theoretical extinction coefficient calculated from sequence by ProtParam was used.[2]

NMR measurements of enzyme diastereoselectivity: NMR spectra were measured on a 500-MHz Bruker Avance III HD spectrometer (^{1}H at 500.0 MHz) equipped with a cryoprobe. 10 µM of FlA1 or StDUf62 with 200 µM of racemic SAM were measured in a 50 mM phosphate buffer (pH 8) with an addition of D_2O (total 10% D_2O) and 15 µM DSS (sodium trimethylsilylpropanesulfonate) as an internal standard. NaF (20 mM) was added in case of FlA1. A series of 1H NMR spectra was measured at 303 K.

LC-MS measurements of enzyme activity: Reactions (triplicate for each condition, 50 µL final volume) were prepared by adding enzyme (10 µM final concentration) into a buffer (50 mM phosphate, pH 8). Depending on running conditions specified, NaCl (150 or 300 mM), KCl (150 or 300 mM), adenosine (300 µM) or sodium fluoride (20 mM) was added. Reactions were initiated by addition of SAM (as freshly dissolved *S*-adenosyl-L-methionine disulfate tosylate in 50 mM phosphate buffer, pH 8, 300 µM final reaction concentration) and were incubated for 15 min at 30 °C. After this, tubes containing reactions were frozen in ethanol/dry ice slurry. Every tube was thawed immediately before a particular LC-MS run by addition of 100 µL of 80% (v/v) ethanol to ensure no delay between reaction end and measurement. Methionine formation, a common feature of both fluorinase and StDUF reaction, was used to monitor reaction progress. LC–MS analyses were performed using a Vanquish Flex UHPLC System interfaced to an Orbitrap ID-X Tribrid mass spectrometer equipped with a heated electrospray ionization (H-ESI) source. The LC conditions were as follows: column─Waters BEH C18, 50 × 2.1 mm, 1.7 µm; mobile phase─(A) water with 0.1% formic acid and (B) acetonitrile with 0.1% formic acid; flow rate─350 µL/min; column oven temperature─40 °C; injection volume─1 µL; isocratic 5% B for 1.5 min, linear gradient from 5 to 60% B over 8 min and isocratic at 5% B for 2 min. ESI was measured in positive mode, and mass spectrometer parameters were as follows: ion transfer tube temperature, 325 °C; auxiliary gas flow rate, 10 (arbitrary units (AU)); vaporizer temperature, 350 °C; sheath gas flow rate, 50 (AU); sweep gas flow rate, 1 (AU); capillary voltage, 3000 V; resolution, 60,000, quadrupole isolation; scan range, *m*/*z* 100–1000; RF Lens, 45%; maximum injection time, 118 ms.

Analytical ultracentrifugation: Sedimentation velocity (SV) experiments were performed using a Optima AUC analytical ultracentrifuge (Beckman Coulter, Brea, CA, USA). SV experiments were conducted in charcoal-filled Epon centerpieces with a 12 mm optical path length at 20 °C and at rotor speeds ranging from 45 000 rpm (163 296 rcf, An-50 Ti rotor, Beckman Coulter). All sedimentation profiles were recorded with interference optics. Buffer density and viscosity were estimated using the program SEDNTERP.[3] Diffusion-deconvoluted sedimentation coefficient distributions c(s) were calculated from raw data using the SEDFIT package.[4]

Description	Sequence (5'→3')
flA1 from *Streptomyces* sp. MA37	ATGGGCAGCAGCCATCATCATCATCATCACAGCAGCGGCGAGAATC<br>TTTATTTTCAGGGCCATGCCGCCAACGGCAGCCAGCGCCCGATCAT<br>CGCGTTCATGTCCGACCTGGGCACCACCGACGACAGCGTGGCGCA<br>GTGCAAGGGCCTGATGCACAGCATCTGCCCCGGTGTGACCGTGGT<br>GGATGTGTGCCACAGCATGACCCCGTGGGACGTCGAGGAGGGCG<br>CCCGTTACATCGTGGACCTGCCGCGTTTCTTCCCGGAGGGCACCG<br>TCTTCGCCACCACCACCTATCCCGCCACCGGCACCACCACCCGCA<br>GCGTGGCCGTGCGTATCCGCCAGGCCGCCAAAGGCGGCGCCCGT<br>GGCCAGTGGGCCGGCAGCGGCGACGGCTTCGAACGTGCCGACG<br>GCAGCTACATCTACATCGCCCCGAACAACGGCCTGCTGACCACCGT<br>GCTGGAGGAACACGGCTATATCGAGGCCTACGAGGTGACCAGCAC<br>CAAGGTGATCCCGGCGAACCCGGAGCCGACCTTCTACAGCCGCGA<br>AATGGTGGCCATCCCGTCCGCCCACCTGGCCGCCGGCTTCCCGCT<br>GGCCGAGGTGGGCCGTCGTCTGGATGACAGCGAGATCGTCCGTTT<br>CCACCGCCCCGCCGTCGAGATCTCCGGCGAAGCCCTGAGCGGCG<br>TGGTGACCGCCATCGACCACCCGTTCGGCAACATCTGGACCAACAT<br>CCACCGTACCGACCTGGAAAAGGCCGGCATCGGCCAGGGCAAACA<br>CCTGAAGATCATCCTGGACGACGTGCTGCCGTTCGAAGCCCCGCT<br>GACCCCCACCTTCGCCGACGCCGGCGCCATCGGCAACATCGCCTT<br>CTACCTGAACAGCCGCGGCTATCTGAGCCTGGCCCGCAACGCCGC<br>CAGCCTGGCCTATCCGTACAACCTGAAAGCCGGCCTGAAGGTGCG<br>CGTGGAGGCCCGTTGA
duf62 from *Salinispora tropica*	ATGGGCAGCAGCCATCATCATCATCATCACAGCAGCGGCCTGGTGC<br>CGCGCGGCAGCCATATGGAAAACCTGTATTTCCAGGGTATGGCACC<br>GACTCCATGGATCAGCTTCACGACCGACTATGGCCTGGCGGATGG<br>CTTTGTTGCGGCATGCCACGGTGTTCTGGCCCGCCTGACCCCAAC<br>TACCCGTGTTATCGACGTTACTCACCTGGTGCCGCCAGGTGATGTT<br>CGTCGTGGTGCAGCGGTGCTGGCTCAGGCTGTGCCGTATCTGCCG<br>GCAGCAGTCCACCTGGCTGTAGTGGACCCAGGCGTTGGTACTGCA<br>CGTCGTGCAATTGCTCTGGCAGCGGGCGATGGTCTGCTGGTAGGT<br>CCAGACAACGGCCTGCTGCTGGATGCAGCTGCAGCTCTGGGTGGT<br>GTGCGTGCGGCTGTAGAACTGACGAACCGTGACTGGCTGGGCGC<br>CGATGTGTCTGCGACCTTCCACGGTCGCGATATCTTTGCGCCGGTT<br>GCTGCCCGCCTGGCGCTGGGTGCACCGCTGGCGGATGCGGGTCC<br>AGCTGTAGAACCAAGCACCCTGGTTCGTCTGCCGGTCCCGCTGGT<br>TCGTCCGGAAGCTGACGGTTTTACCGCGGAGGTGCTGACCGTTGA<br>CCACTTCGGCAACGTTCAGCTGGCCGCCAGCGGTTCCCTGCTGGA<br>ACCGCTGCCGCGTTCTCTGCGTGTTGAACGTCAGCCGGCTGTGCG<br>TGTACACACCTTCGGTGACGTGGCGCCGGGTGAGCTGCTGGTACA<br>CGTAGATTCTACCGGTCAGGTCGCGGTAGCGGTAAATGGTGGTCGC<br>GCTGCTGATCTGCTGGGTGTAACCCCGGGCGATCGTCTGCGCGTT<br>ACCGCCGGCTAA

Table S1: DNA sequences of proteins studied, including N-terminal His-tag and TEV site

Description	Sequence
FIA1 from *Streptomyces* sp. MA37	MGSSHHHHHHSSGENLYFQGHAANGSQRPIIAFMSDLGTTDDSVAQC<br>KGLMHSICPGVTVVDVCHSMTPWDVEEGARYIVDLPRFFPEGTVFATT<br>TYPATGTTTRSVAVRIRQAAKGGARGQWAGSGDGFERADGSYIYIAPN<br>NGLLTTVLEEHGYIEAYEVTSTKVIPANPEPTFYSREMVAIPSAHLAAGF<br>PLAEVGRRLDDSEIVRFHRPAVEISGEALSGVVTAIDHPFGNIWTNIHRT<br>DLEKAGIGQGKHLKIILDDVLPFEAPLTPTFADAGAIGNIAFYLNSRGYL<br>SLARNAASLAYPYNLKAGLKVRVEAR
StDUF62 from *Salinispora tropica*	MGSSHHHHHHSSGLVPRGSHMENLYFQGMAPTPWISFTTDYGLADG<br>FVAACHGVLARLTPTTRVIDVTHLVPPGDVRRGAAVLAQAVPYLPAAVH<br>LAVVDPGVGTARRAIALAAGDGLLVGPDNGLLLDAAAALGGVRAAVEL<br>TNRDWLGADVSATFHGRDIFAPVAARLALGAPLADAGPAVEPSTLVRL<br>PVPLVRPEADGFTAEVLTVDHFGNVQLAASGSLLEPLPRSLRVERQPA<br>VRVHTFGDVAPGELLVHVDSTGQVAVAVNGGRAADLLGVTPGDRLRV<br>TAG

Table S2: Amino acid sequences of proteins studied, including N-terminal His-tag and TEV site

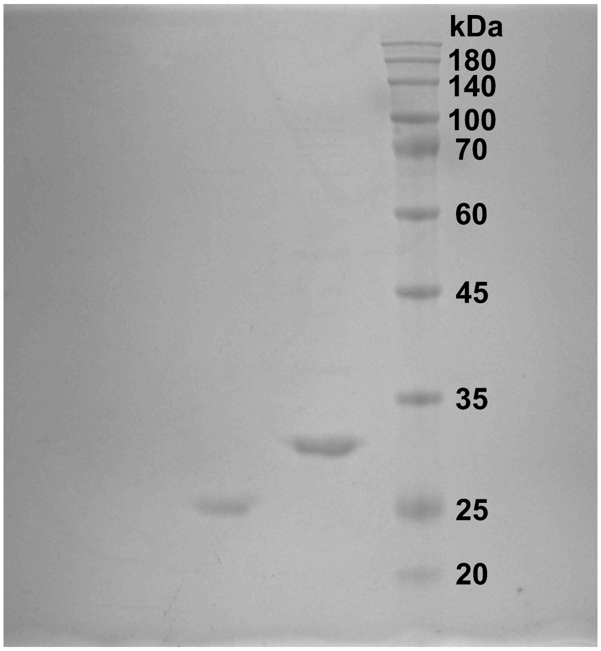

Figure S1: 12% SDS-PAGE gel of the proteins studied. From left to right, StDUF62 (26.5 kDa), FIA1 (32.2 kDa) and ROTI®Mark TRICOLOR marker

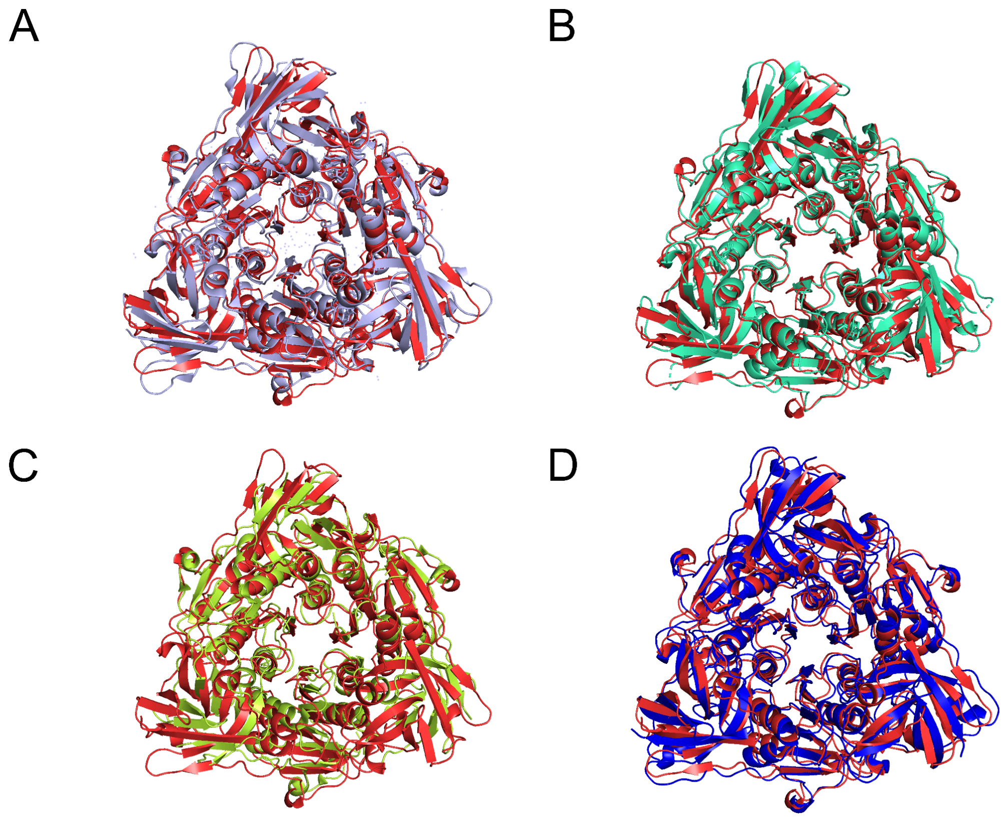

Figure S2: 3D alignment of published structures of proteins from DUF62 family and predicted structure of StDUF62 from AlphaFold (red; AF DB: AF-A4X4S2-F1-v4). A) *Streptomyces noursei* DUF62 (PDB ID: 7ccg)[5] aligned on StDUF62 (47.8% sequence similarity and 33.8% sequence identity). B) *Thermotoga maritima* DUF62 (PDB ID: 2zbv)[6] aligned on StDUF62 (52.5% sequence similarity and 38.4% sequence identity). C) *Thermus thermophilus* DUF62 (PDB ID: 2cw5)[7] aligned on StDUF62 (51.6% sequence similarity and 42.5% sequence identity). D) Fluorinase from *Streptomyces* sp. MA 37 (PDB ID: 5b6i)[8] aligned on StDUF62 (42.3% sequence similarity and 27.7% sequence identity). Molecular graphics were rendered with PyMOL 2.5.8.

Supplementary references

1. Pardo, I. *et al.* A Nonconventional Archaeal Fluorinase Identified by In Silico Mining for Enhanced Fluorine Biocatalysis. *ACS Catalysis*, **12**, 11, 6570–6577. (2022)

2. Walker, J. M. *The Proteomics Protocols Handbook*. (Humana Press).
3. Philo, J. S. SEDNTERP: a calculation and database utility to aid interpretation of analytical ultracentrifugation and light scattering data. *Eur. Biophys. J.* **52**, 233–266 (2023).
4. Schuck, P. Size-distribution analysis of macromolecules by sedimentation velocity ultracentrifugation and lamm equation modeling. *Biophys. J.* **78**, 1606–1619 (2000).

5. Miao, Y., Yu, J., Ouyang, Z., Sun, H., Li, Y. Crystal structure of ClA1, a type of chlorinase from soil bacteria, *Biochemical and Biophysical Research Communications*. **530**, 1, 42–46 (2020).

6. Ebihara A, Fujimoto Y, Kagawa W, Fujikawa N, Chen L, Fu ZQ, Chrzas J, Wang BC, Yokoyama S, Kuramitsu S, RIKEN Structural Genomics/Proteomics Initiative (RSGI), Crystal structure of uncharacterized conserved protein from Thermotoga maritima (2007) https://doi.org/10.2210/pdb2zbv/pdb

7. Ebihara A, Yokoyama S, Kuramitsu S, RIKEN Structural Genomics/Proteomics Initiative

(RSGI), Crystal structure of a conserved hypothetical protein from Thermus thermophilus HB8 (2005) https://doi.org/10.2210/pdb2cw5/pdb

8. Sun, H. *et al.* Directed Evolution of a Fluorinase for Improved Fluorination Efficiency with a Non-native Substrate. *Angew. Chem. Int. Ed Engl.* **55**, 14277–14280 (2016).
